# The Nutrition Knowledge of Croatian General Practitioners

**DOI:** 10.3390/jcm7070178

**Published:** 2018-07-19

**Authors:** Albina Dumic, Maja Miskulin, Nika Pavlovic, Zelimir Orkic, Vesna Bilic-Kirin, Ivan Miskulin

**Affiliations:** Faculty of Medicine Osijek, Josip Juraj Strossmayer University of Osijek, Osijek 31000, Croatia; albina.dumic@gmail.com (A.D.); maja.miskulin@mefos.hr (M.M.); nika.pavlovic@mefos.hr (N.P.); zelimir.orkic@gmail.com (Z.O.); vesna.bilic.kirin@gmail.com (V.B.-K.)

**Keywords:** nutrition, knowledge, general practitioners, primary health care, Croatia

## Abstract

Nutrition care delivered in primary health care setting is an effective and necessary preventive health care measure. General practitioners (GPs) nutrition knowledge is related to their nutrition care practice. The aim of this study was to explore the nutrition knowledge of Croatian GPs, and to investigate its connection with the implementation of nutrition care in GPs’ offices. A cross-sectional study was conducted among 17.0% of randomly selected GPs, from May to July 2013, via an anonymous questionnaire. The study showed that only 35.8% of the Croatian GPs had an adequate level of nutrition knowledge (five or more correct answers to nutrition questions). The study further revealed that females, GPs with additional education in nutrition and GPs who had not suffered from chronic diseases with poor nutrition posing as a risk factor had better nutrition knowledge (*p* = 0.029, *p* < 0.001 and *p* = 0.041, respectively). The Spearman rank correlation between GPs’ nutrition knowledge and the implementation of nutrition care in their offices during daily work with patients was *r_s_* = −0.190 (*p* < 0.001). To provide nutrition care in GPs’ offices in Croatia, strategies for improving GPs’ nutrition knowledge are needed.

## 1. Introduction

Nutrition care is an important component of healthcare that plays a critical role in the prevention and treatment of majority of chronic non-communicable diseases which are leading causes of morbidity and mortality throughout the world [1,2]. Nutrition care refers to any practice undertaken by a health professional to improve an individual’s food-related behavior and subsequent health outcomes [3].

General practitioners (GPs) are ideally placed within the healthcare system to provide nutrition care to their patients [4]. Nutrition care delivered in primary health care setting is an effective and necessary preventive health care measure for the prevention of the chronic non-communicable diseases [5]. Moreover, health workers in primary health care settings are particularly important providers of nutrition care because they can motivate even healthy individuals to adopt healthier lifestyles [2,3].

Nutrition knowledge is one of the factors that affect the nutritional habits of individuals, families and communities [6]. The nutrition care practices of physicians in the primary care setting is strongly influenced by their nutrition knowledge [7,8,9,10]. The nutritional knowledge of Croatian GPs is unknown. Thus, the aim of this study was to explore the knowledge of Croatian GPs about nutrition, and to evaluate the interconnection between their knowledge and implementation of nutrition care in GPs’ offices.

## 2. Materials and Methods

### 2.1. Study Population and Survey

This cross-sectional study was conducted from 1 May to 31 July 2013. The study was approved by the Ethics Committee of the Faculty of Medicine Osijek, Croatia (Ethical Approval Code: 2158-61-07-12-35).

A random selection of doctors who work in primary care in Croatia was made from the register of GPs managed by the Croatian National Institute of Public Health. Eight hundred Croatian GPs (30.6% of GPs) were mailed a description of the study, a consent form and the anonymous questionnaire. They were asked to sign the consent form before answering the questionnaire and to put the signed informed consent form in one envelope, and the answered questionnaire in the other envelope and then send them to the lead author. The answered questionnaires that arrived via post were coded, and the data provided within the questionnaires were later analyzed using that code.

### 2.2. Questionnaire

This anonymous self-administered questionnaire consisted of 30 questions divided into four sections. The first section of the questionnaire (six questions) referred to the sociodemographic characteristics of the study participants such as age, gender, education, length of service, additional education in the field of nutritional science, and ailments from chronic diseases where poor nutrition poses as a risk factor. The second section of the questionnaire (ten closed questions with four offered answers and only one is correct) assessed GP’s nutrition knowledge. The third section of the questionnaire (ten closed questions with “agree/do not agree” answers) involved the attitudes the GPs had toward the significance of the nutrition in the treatment and prevention of chronic diseases and toward the nutrition care. The fourth section of the questionnaire (four closed questions with offered answers) referred to the nutrition care of GPs in their daily work with patients. The questionnaire used in this study was previously validated with a smaller group of participants in 2012. The first version of the questionnaire had 26 questions divided into four sections. The first draft of the questionnaire was reviewed by a panel of professionals which consisted of two nutritionists, one family medicine professor and one epidemiology professor with experience in a survey-type research. Before the distribution, this modified version was pilot tested among 40 GPs in Health Center Osijek, Croatia to assess the content and validity of the research tool. In concordance to reliability analysis, the final version of the questionnaire had 30 questions. Within this manuscript, questions about the sociodemographic characteristics of the study participants, questions about the GPs’ nutrition knowledge as well as question regarding the implementation of nutrition care in the GPs’ everyday work with patients were analyzed. For the analysis of a satisfactory number of correct answers, a cut-off value of five correct answers was applied, because it has been shown that correct answers of 50% or more are sufficient to ensure acceptable nutrition care in GPs offices [11].

### 2.3. Statistical Analysis

Following confirmation of the normality of the data distribution by the Kolmogorov–Smirnov test, all data were processed by the methods of descriptive statistics. The numerical variables were described as median and interquartile range. The Mann–Whitney U test was used for comparison of numerical variables among the groups. The categorical variables were described in absolute and relative frequencies. The χ^2^-test was used for the comparison of categorical variables between the groups. The Spearman’s correlation coefficient (*r_s_*) was calculated to test the correlation between the implementation of nutrition care in GPs’ everyday work with patients and their nutrition knowledge. The level of statistical significance was set at *p* < 0.05. Statistical analysis was done using the statistical package Statistica for Windows 2010 (version 10.0, StatSoft Inc., Tulsa, OK, USA).

## 3. Results

The response rate was 55.5% (444/800). The study included 17.0% (444/2.612) of doctors who work in primary health care offices in Croatia, and the study participant sample was representative for this population of Croatian doctors. The sample was 81.3% females and 18.7% males. The mean age of participants was 50 years (range 25–67 years). The majority of doctors were specialists in family medicine (67.3%) and a minority of doctors (32.7%) had licenses for an independent practice but had not completed specialization in family medicine. Most participant doctors (65.1%) had ≥15 years in practice while 34.9% participants had 0–14 years of service. A minority of participants (9.5%) had completed additional educational program in the field of nutritional science and almost a third (30.6%) suffered from a chronic disease. Over one third of participants (35.8%) answered five or more nutrition knowledge questions correctly and 64.2% of them answered only four or fewer questions correctly.

Table 1 shows nutrition knowledge questions with correct answers and percentage of Croatian GPs who gave correct answer.

Table 2 shows Croatian GPs according to their sociodemographic characteristics and the number of correct answers to nutrition questions.

The median number of correct answers to questions about nutrition knowledge for Croatian GPs was 4.00 (interquartile range 3.00–6.00).

Table 3 shows Croatian GPs nutrition knowledge according to the sociodemographic characteristics of study participants.

The study showed that, during their everyday work with patients, 95.7% of Croatian GPs provide nutrition care, while 4.3% of them do not provide such care at all. This study demonstrated that the majority of Croatian GPs (80.5%) who provide such care only for patients considered at risk with regard to their poor nutrition and/or elevated body mass index while 19.5% of GPs provide nutrition care for all patients regardless of their individual health risks. The Spearman rank correlation between GPs knowledge about nutrition and their practice, i.e., the implementation of nutrition care in GPs everyday work with patients, was *r_s_* = −0.190 (*p* < 0.001).

## 4. Discussion

In this study, we assessed Croatian GPs’ nutrition knowledge and implementation of nutrition care of their everyday work with patients. The study showed that only 35.8% of Croatian GPs had a satisfactory number of correct answers (5 or more) to nutrition knowledge questions. This is of concern related to the rather unsatisfactory situation regarding the researched issues within the study population. These results differ from the results of similar studies conducted in Saudi Arabia, Taiwan, Kuwait, Qatar and the UK, where the percentage of correct answers to questions about nutrition were 52.1%, 59%, 60%, 64% and 65%, respectively [12,13,14,15,16].

Some areas of nutrition are better known to Croatian GPs such as nutrient believed to prevent thrombosis (74.5% of correct answers), nutrient strongly associated with the prevention of neural tube defects (86.0% of correct answers) and the value of body mass index (BMI) that indicates obesity (81.6% of correct answers). This is similar to the study conducted in Qatar where the best known item (answered correctly at the highest rate) by GPs is that folate is the nutrient strongly associated with the prevention of neutral tube defect [15]. However, some parts of nutrition science seem to be extremely unknown to Croatian GPs, for example, the most concentrated source of vitamin B12 (only 14.0% or correct answers) or the major type of fat in olive oil (26.2% of correct answers). Percentages of correct answers to all other nutrition knowledge questions are somewhere in between these two groups. Such findings point to the fact that Croatian GPs have very poor knowledge of general nutrition while they have better knowledge of some parts of clinical nutrition. These findings were opposite to the results of similar study from Taiwan where GPs scored higher on general nutrition knowledge questions [13]. A possible explanation for this finding could be the fact that curricula of Croatian medical faculties and modern lifetime education of GPs tend to incorporate nutritional knowledge connected with the possibility of treatment and prevention of some diseases through diet improvement.

The present study showed that there was no statistically significant difference in the proportion of Croatian GPs with and without a satisfactory number of correct answers about nutrition between GPs with ≥14 and GPs with ≥15 years of experience, between younger GPs (≤45 years old) and older GPs (≥46 years old), or between specialists of family medicine and GPs without specialization. In a study from Kuwait, it was found that there was no statistically significant difference in nutrition knowledge levels between younger and older GPs, which is in accordance with this study [14]. However, some studies conducted elsewhere in the world showed different results. For example, in the study conducted in Taiwan nutritional knowledge scores were significantly higher for younger physicians (under 35 years of age) and physicians with less years of experience [13]. In a study from Iran, it was found that older physicians with more years of experience tend to have poorer nutrition knowledge than younger ones with less year of experience [17].

The present study further revealed that female GPs, GPs with additional education in nutrition and GPs who had not suffered from chronic diseases with improper nutrition posing as a risk factor had better nutrition knowledge. Female doctors having better nutrition knowledge in this study is in accordance with the results of studies conducted among GPs in Taiwan and Kuwait where females also had significantly higher levels of knowledge [13,14]. The better nutrition knowledge found in females can be connected to the fact that females in general are more concerned about their nutrition and health than males [18]. The connection between additional education in nutrition and better nutrition knowledge is logical and understandable by itself, although there are some studies that point to the necessity of transforming nutrition education for physicians to achieve even better nutrition knowledge [19,20,21,22,23,24,25]. It has been emphasized that improved education in nutrition is essential at all levels of physician training. It is further expected that with better education physicians can become more effective at providing the nutrition care for their patients [19].

Regarding the personal ailment from chronic diseases and nutrition knowledge, it seems that Croatian GPs who have suffered from chronic diseases with improper nutrition posing as a risk factor, despite their illness, still do not recognize the significance of nutritional factors in their etiology. Such finding was also determined for the interconnection between the personal ailment from chronic diseases and attitudes toward the significance of nutrition in the treatment and prevention of chronic diseases and toward nutrition care among Croatian GPs [3]. GPs with poor nutrition knowledge that suffer from some kind of chronic diseases and despite that do not recognize the significance of nutritional factors in etiology of their diseases are bad role models for their patients. This can represent serious obstacles in delivery of nutrition care within the primary health care setting in Croatia because it is well known that those physicians who modified their own nutrition and know more about nutrition were more likely to apply nutrition care in their practice.

The key finding of this study is that a minority of Croatian GPs (only 35.8%) answered the nutrition knowledge questions correctly, yet the majority of them provide nutrition care to their patients (95.7%). This suggests that lack of nutrition knowledge was not a barrier for the nutrition care implementation in GPs offices. The finding about a lack of nutrition knowledge among Croatian GPs is similar to the situation in other European countries, USA and Canada [8,26,27,28,29,30,31]. Although some studies have shown that increased nutritional knowledge seems to improve the nutritional practice [32,33], this study did not show such connection.

The findings of this study significantly increase our understanding regarding the Croatian GPs’ nutrition knowledge. Our evaluation is based on the nationally representative sample of Croatian GPs, with a high representation of different age cohorts of GPs. As previously emphasized, 17.0% of doctors who work in primary health care offices in Croatia had been questioned for this study, so their answers can be considered representative. Several study limitations deserve to be noted. First, its cross-sectional design makes it difficult to establish causality. Nonetheless, it gives a clear snapshot of the Croatian GPs nutrition knowledge. Second, as with all self-administered studies, the present study was subject to volunteer bias, although the effects of this are minimized by the response rate of nearly 60%.

Future studies should explore factors contributing to the current status of Croatian GPs’ nutrition knowledge, especially those connected with the current and future state of medical education in Croatia, both, within the curricula of medical faculties in Croatia and within the continuous medical education for physicians.

## 5. Conclusions

The proportion of Croatian GPs with satisfactory level of nutrition knowledge is currently not very high. There is no interconnection between GPs’ nutrition knowledge and implementation of nutrition care in their offices. The findings of this study also indicate that, as with the international literature, Croatian GPs lack nutrition knowledge. To increase the proportion of Croatian GPs with adequate level of nutrition knowledge, and also to encourage the Croatian GPs in their nutrition care practices, it is necessary to design and implement health policy measures that are predominantly oriented toward prevention.

## Figures and Tables

**Table 1:** Nutrition knowledge questions with correct answers and percentage of Croatian GPs who gave correct answer.

Questions Asked	Correct Answer	Percent of Croatian GPs Who Gave Correct Answer
A nutrient believed to help prevent thrombosis	Omega-3 fat	74.5%
The average daily intake of salt in Croatian adult population	11–14 grams	33.6%
The major type of fat in olive oil	Monounsaturated fat	23.2%
Compared with unprocessed vegetable oil, hydrogenated fats contain	More trans fats	28.6%
The most concentrated source of vitamin B_12_	Meat	14.0%
Type of food believed to have a preventive effect on various types of cancer	Fruit and vegetable	39.0%
Which of the following is not an antioxidant nutrient	Zinc	23.6%
Which nutrient is usually lacking in people who drink alcohol excessively	Vitamin B_1_ (tiamin)	40.1%
The nutrient strongly associated with the prevention of neural tube defects	Folate	86.0%
The body mass index (BMI) value that indicates obesity	BMI ≥ 30	83.6%

**Table 2:** Croatian GPs with and without a satisfactory number of correct answers to nutrition questions according to their sociodemographic characteristics.

Participant Characteristics	Number of Participants (%)			*p* *
	With Satisfactory Number of Correct Answers (5 or More)	Without Satisfactory Number of Correct Answers (4 or Less)	Overall	
**Gender**				
Male	22 (13.8)	61 (21.4)	83 (18.7)	0.057
Female	137 (86.2)	224 (78.6)	361 (81.3)	
**Length of service (years)**				
0–14	55 (34.6)	100 (35.1)	155 (34.9)	0.918
15 or more	104 (65.4)	185 (64.9)	289 (65.1)	
**Age group (years)**				
Younger (45 or less)	59 (37.1)	107 (37.5)	166 (37.4)	1.000
Older (46 or more)	100 (62.9)	178 (62.5)	278 (62.6)	
**Education**				
Doctors with a license for independent practice without a finished specialization	55 (34.6)	90 (31.6)	145 (32.7)	0.528
Specialist in family medicine	104 (65.4)	195 (68.4)	299 (67.3)	
**Additional education about nutrition**				
Yes	32 (20.1)	10 (3.5)	42 (9.5)	<0.001
No	127 (79.9)	275 (96.5)	402 (90.5)	
**Suffering from chronic diseases**				
Yes	35 (22.0)	101 (35.4)	136 (30.6)	0.004
No	124 (78.0)	184 (64.6)	308 (69.4)	
**Overall**	159 (35.8)	285 (64.2)	444 (100.0)	

* χ^2^-test.

**Table 3:** Croatian GPs nutrition knowledge according to the sociodemographic characteristics of study participants.

Participant Characteristics	Number of Correct Answers (%)		*p* *
	Median (25–75%)	Min–Max	
**Gender**			
Male	4.00 (3.00–5.00)	1.00–10.00	0.029
Female	4.00 (3.00–6.00)	0–10.00	
**Length of service (years)**			
0–14	4.00 (3.00–6.00)	0–10.00	0.498
15 or more	4.00 (3.00–6.00)	1.00–10.00	
**Age group (years)**			
Younger (45 or less)	4.00 (3.00–6.00)	0–10.00	0.446
Older (46 or more)	4.00 (3.00–6.00)	1.00–10.00	
**Education**			
Doctors with a license for independent practice without a finished specialization	4.00 (3.00–6.00)	1.00–10.00	0.433
Specialist in family medicine	4.00 (3.00–6.00)	0–10.00	
**Additional education about nutrition**			
Yes	6.00 (5.00–7.00)	2.00–10.00	<0.001
No	4.00 (3.00–5.00)	0–10.00	
**Suffering from chronic diseases**			
Yes	4.00 (3.00–5.00)	1.00–10.00	0.041
No	4.00 (3.00–6.00)	0–10.00	
**Overall**	4.00 (3.00–6.00)	0–10.00	

* Mann–Whitney U test.

## References

[1] Crowley J., Ball L., Wall C. (2016). Nutrition advice provided by general practice registrars: An investigation using patient scenarios. Public Health.

[2] Mogre V., Scherpbier A.J.J.A., Stevens F., Aryee P., Cherry M.G., Dornan T. (2016). Realist synthesis of educational interventions to improve nutrition care competencies and delivery by doctors and other healthcare professionals. BMJ Open.

[3] Dumic A., Miskulin I., Pavlovic N., Cacic Kenjeric D., Orkic Z., Miskulin M. (2018). Attitudes toward Nutrition Care among General Practitioners in Croatia. J. Clin. Med..

[4] Dumic A., Miskulin I., Matic Licanin M., Mujkic A., Cacic Kenjeric D., Miskulin M. (2017). Nutrition Counselling Practices among General Practitioners in Croatia. Int. J. Environ. Res. Public Health.

[5] Ferrari Bravo M., Gallo F., Marchello C., Boicelli R., Lupi S., Atzei M., Brunetti F., Casaretto R., Dapelo F., Gerevini D. (2018). Assessment of Malnutrition in Community-dwelling Elderly People: Cooperation among General Practitioners and Public Health. Iran. J. Public Health.

[6] Uddin M.T., Islam M.N., Uddin M.J. (2008). A survey on knowledge of nutrition of physicians in Bangladesh: Evidence from Sylhet data. South East Asian J. Med. Educ..

[7] Truswell A.S., Hiddink G.J., Blom J. (2003). Nutrition guidance by family doctors in a changing world: Problems, opportunities, and future possibilities. Am. J. Clin. Nutr..

[8] Mowe M., Bosaeus I., Rasmussen H.H., Kondrup J., Unosson M., Rothenberg E., Irtun Ø., Scandinavian Nutrition Group (2008). Insufficient nutritional knowledge among health care workers?. Clin. Nutr..

[9] Al-Muammar M.N. (2012). Predictors of physicians’ practices related to nutritional counseling and management in Riyadh City. Alex. J. Med..

[10] Nowson C.A., O’Connell S.L. (2015). Nutrition Knowledge, Attitudes, and Confidence of Australian General Practice Registrars. J. Biomed. Educ..

[11] Kołłajtis-Dołowy A., Żamojcin K. (2016). The level of knowledge on nutrition and its relation to health among Polish young men. Rocz. Państwowego Zakl. Hig..

[12] Al-Zahrani A.M., Al-Raddadi R.M. (2009). Nutritional knowledge of primary health care physicians in Jeddah, Saudi Arabia. Saudi Med. J..

[13] Hu S.P., Wu M.Y., Liu J.F. (1997). Nutrition knowledge, attitude and practice among primary care physicians in Taiwan. J. Am. Coll. Nutr..

[14] Allafi A.R., Alajmi F., Al-Haifi A. (2013). Survey of nutrition knowledge of physicians in Kuwait. Public Health Nutr..

[15] Daradkeh G.A.F., Al Bader K., Singh R. (2012). The Nutrition Knowledge of Primary Care Physicians in the State of Qatar. Pak. J. Nutr..

[16] Moore H., Adamson A.J. (2002). Nutrition interventions by primary care staff: A survey of involvement, knowledge and attitude. Public Health Nutr..

[17] Ahmadi A., Ershad M., Givzadeh H., Mohammad-Beigi A. (2009). General physicians’ knowledge about nutrition in Shiraz, Iran. Pak. J. Biol. Sci..

[18] Lupi S., Bagordo F., Stefanati A., Grassi T., Piccinni L., Bergamini M., De Donno A. (2015). Assessment of lifestyle and eating habits among undergraduate students in northern Italy. Ann. Ist. Super. Sanita.

[19] Azizi M., Rahmani-Nia F., Malaee M., Malaee M., Khosravi N. (2010). A study of nutritional knowledge and attitudes of elite college athletes in Iran. Braz. J. Biomot..

[20] Gies I., AlSaleem B., Olang B., Karima B., Samy G., Husain K., Elhalik M., Miqdady M., Rawashdeh M., Salah M. (2017). Early childhood obesity: A survey of knowledge and practices of physicians from the Middle East and North Africa. BMC Pediatr..

[21] Bucher Della Torre S., Courvoisier D.S., Saldarriaga A., Martin X.E., Farpour-Lambert N.J. (2018). Knowledge, attitudes, representations and declared practices of nurses and physicians about obesity in a university hospital: Training is essential. Clin. Obes..

[22] Lee B., Trence D., Inzucchi S., Lin J., Haimowitz S., Wilkerson E., Williams C., Mosier M., Dex T. (2016). Improving Type 2 Diabetes Patient Health Outcomes with Individualized Continuing Medical Education for Primary Care. Diabetes Ther..

[23] Martin L., Leveritt M.D., Desbrow B., Ball L.E. (2014). The self-perceived knowledge, skills and attitudes of Australian practice nurses in providing nutrition care to patients with chronic disease. Fam. Pract..

[24] Al-Najjar A.A., Al-Jasem N.J.M., Al-Quraini Y.F., Salama O., El-Shazly M.K. (2012). Knowledge and attitude of primary health care doctors towards obesity management, Kuwait. Greener J. Med. Sci..

[25] Fogelman Y., Vinker S., Lachter J., Biderman A., Itzhak B., Kitai E. (2002). Managing obesity: A survey of attitudes and practices among Israeli primary care physicians. Int. J. Obes. Relat. Metab. Disord..

[26] Vetter M.L., Herring S.J., Sood M., Shah N.R., Kalet A.L. (2008). What do resident physicians know about nutrition? An evaluation of attitudes, self-perceived proficiency and knowledge. J. Am. Coll. Nutr..

[27] Leslie F.C., Thomas S. (2009). Competent to care. Are all doctors competent in nutrition?. Proc. Nutr. Soc..

[28] Wynn K., Trudeau J.D., Taunton K., Gowans M., Scott I. (2010). Nutrition in primary care: Current practices, attitudes, and barriers. Can. Fam. Phys..

[29] Crowley J., Ball L., Han D.Y., McGill A.T., Arroll B., Leveritt M., Wall C. (2015). Doctors’ attitudes and confidence towards providing nutrition care in practice: Comparison of New Zealand medical students, general practice registrars and general practitioners. J. Prim. Health Care.

[30] Crowley J., O’Connell S., Kavka A., Ball L., Nowson C.A. (2016). Australian general practitioners’ views regarding providing nutrition care: Results of a national survey. Public Health.

[31] WHO Regional Office for Europe (2016). Integrating Diet, Physical Activity and Weight Management Services into Primary Care.

[32] Abdollahi M., Houshiarrad A., Abtahi M., Esmaeli M., Pouraram H., Khoshfetrat M.R., Shakori M.M., Keshel S.H. (2013). The nutrition knowledge level of physicians, nurses and nutritionists in some educational hospitals. J. Paramed. Sci..

[33] Mowe M., Bosaeus I., Rasmussen H.H., Kondrup J., Unosson M., Irtun Ø. (2006). Nutritional routines and attitudes among doctors and nurses in Scandinavia: A questionnaire based survey. Clin. Nutr..

